# Coronary angiography causes Rumpel-Leede symptoms

**DOI:** 10.1186/s12872-022-02767-7

**Published:** 2022-07-25

**Authors:** Yang Gui, JingYa Wang, Lifang Ye, GuangXin Pen, Lihong Wang

**Affiliations:** 1 BengBu Medical College, Bengbu, China; 2 Department of Cardiovascular Medicine, Zhejiang Provincial People’s Hospital, People’s Hospital of Hangzhou Medical College, Hangzhou, China; 3 The Second Clinical Medical College, Zhejiang Chinese Medical University, Hangzhou, China

**Keywords:** Rumpel-Leede sign, Coronary angiography, Clinical presentation

## Abstract

**Background:**

Rumpel Leede sign (RLS) is a clinical presentation observed at the extremities due to pressure applied externally. The appearance ranges from scattered pin-point rashes to an entire arm covered with petechial hemorrhage depending upon the severity. This phenomenon is relatively uncommon in clinical practice.

**Case presentation:**

A 64 year old female patient developed a rash in the normal skin area below the compression area on the second day of single catheter coronary angiography. The patient's rash resolved without treatment after 3 days.

**Conclusions:**

We report a case of hypertension and hyperlipidemia with a petechial rash on the skin under the tourniquet compressed by the radial artery after coronary angiography, which is consistent with the Rumpel-Leede phenomenon. clinicians should be watchful of these symptoms.

## Background

This was a typical case of Rumpel-Leede phenomenon. Considering that hypertension is a predisposing factor, persistent radial compression may have caused the venous return to be obstructed, while arterial flow remained normal. Pressurization caused the patient's capillaries to rupture into the dermis, causing a petechial rash.

## Case presentation

**Fig. 1:**
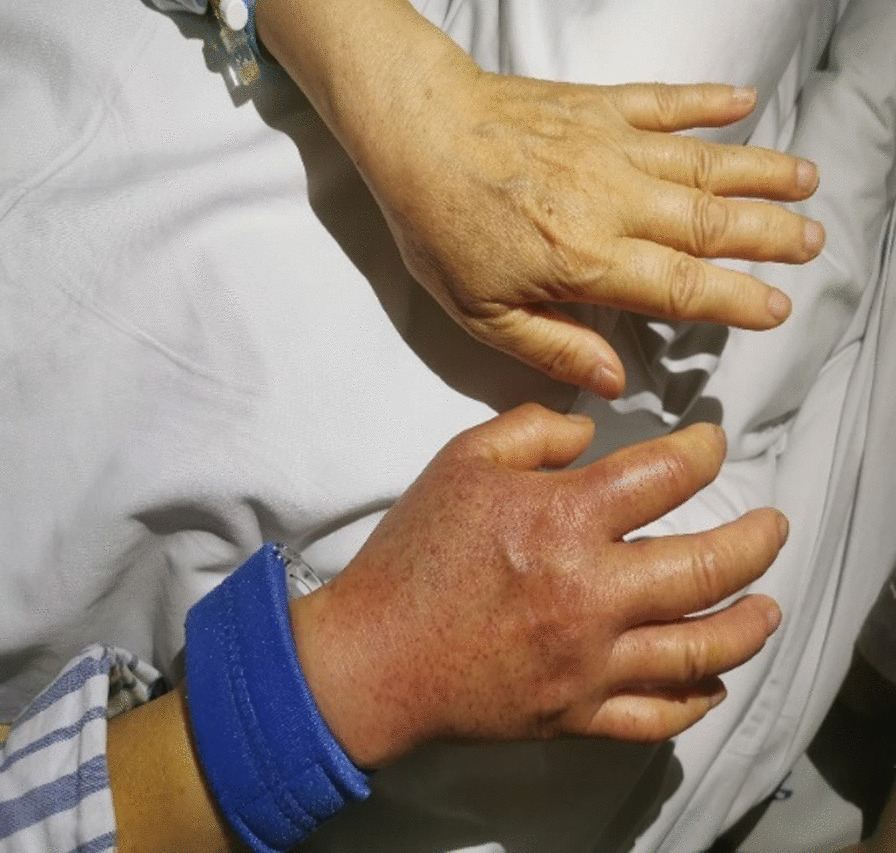
Rumpel-Leede Sign (Hands contrast)

**Fig. 2:**
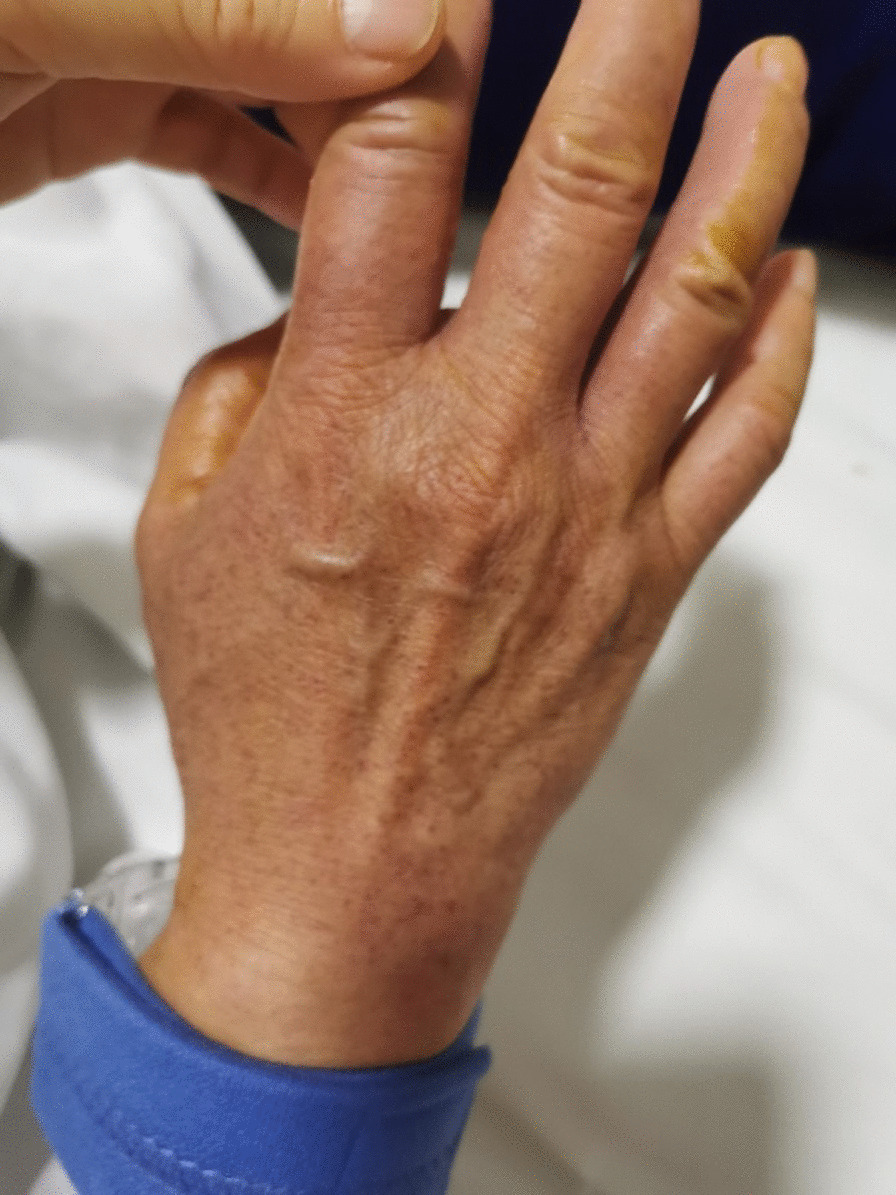
Rumpel-Leede Sign

**Fig. 3:**
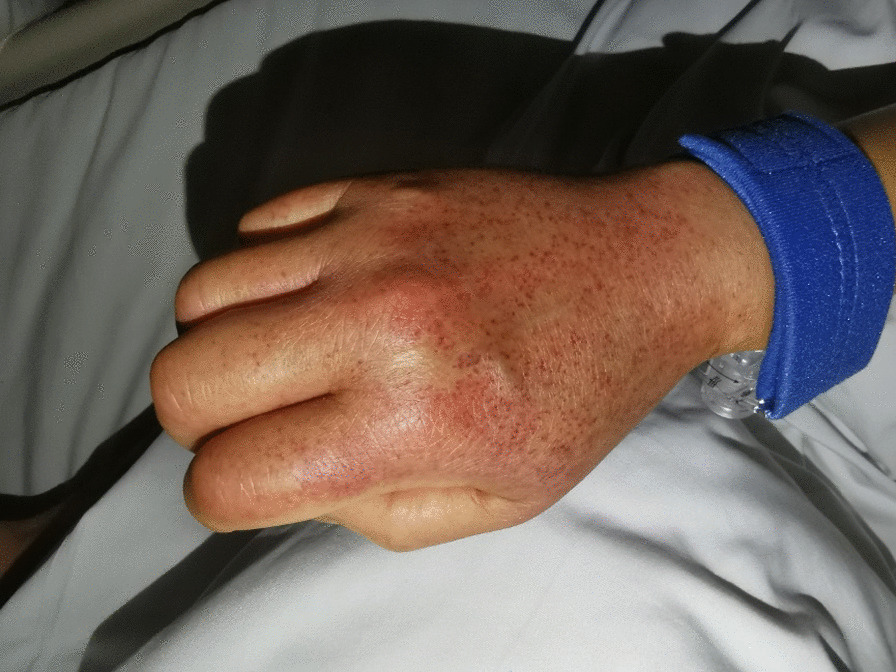
Rumpel-Leede Sign

**Fig. 4:**
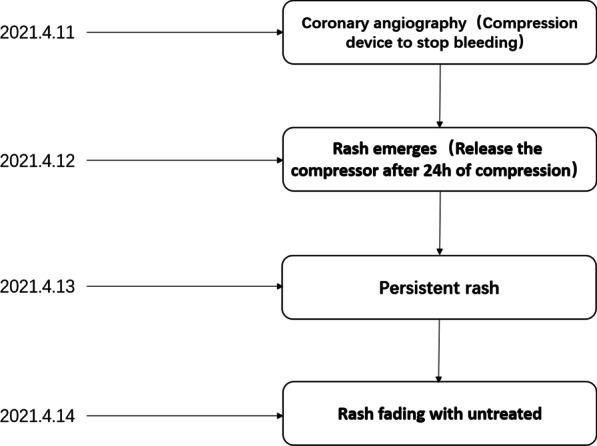
The rash timeline

A 64-year-old female patient presented to the hospital for single-catheter coronary angiography due to persistent chest tightness in the precordial region. The 5F left and right coronal contrast catheters were placed transthecally to allow coronary angiography at multiple projection angles. Postoperatively, the right radial artery was routinely compressed for 24 h using a radial artery compression hemostat (WORK) to stop the bleeding. On the day following the procedure, the patient experienced no redness, swelling, fever, or pain on the right limb; however, the patient had a rash on the right upper limb (Figs. 1, 2, 3). The rash was red, wrinkled, not raised, and well-circumscribed. Ultrasound Doppler examination of the hand was not performed while the rash was present. The patient had a 6-year history of hypertension and previous regular use of Candesartan cilexetil. The patient previously had hyperlipidemia and regularly used Atorvastatin, with normal platelet count, prothrombin time, and activated partial thromboplastin clotting time (APTT) values. The patient's arterial pulsatility was normal. At symptom onset, the patient had a blood pressure of 190/120 mmHg and a platelet count of 167 × 10^9^/L. The patient's rash resolved without treatment after 3 days. The rash timeline is shown in Fig. 4.

## Discussion

This rare condition is generally considered to be caused by a blockage of venous return by compression, with a normal arterial flow, where the patient's capillaries rupture into the dermis, resulting in a petechial rash. Long-term hypertension and high blood lipid levels in patients are susceptibility factors. Rumpel-Leede symptoms in this patient might also have manifested due to aspirin-induced platelet dysfunction and clopidogrel therapy following the coronary angiography. Thus, we have selected and compared several types of acute skin diseases with relatively similar symptoms (Table 1).

**Table 1:** Characteristics of several common acute skin diseases

Diseases	Erythema multiforme	Systemic lupus erythematosus	Acute eczema	Contact dermatitis	Thrombocytopenic purpura
Rash area	Ends of the extremities	Both cheeks, extremities, behind the ears	Face, ears, hands, feet, forearms, and lower legs	Ends of the extremities	Ends of the extremities
Rash features	Initially a well-defined red rash that becomes a raised, edematous papule	Dark purplish red rash	Dense corn-sized papules poorly defined borders	Edematous erythema, papules	Mucosal purpura of skin
Associated symptoms	None	Raynanud’s phenomenon, with joint pain, hair loss and weakness	None	None	Chills and fever, extensive and severe skin, mucosal and visceral bleeding
Test indicators	None	Positive antinuclear antibody, decreased white blood cells, decreased complement C3 and C4	None	None	Low platelet count and prolonged bleeding time

Reports of petechial arm rashes distal to the cuff following both Rumpel tourniquet application in patients with scarlet fever and Leede's tourniquet application have been described by Wang et al. [1]. However, no patient-specific testing parameters were reported. In the case reported by Dubach et al. [2], where blood pressure was measured the day before, and the patient produced multiple petechiae, the patient's platelet count was 38 × 10^9^/L and white blood cells 16 × 10^9^/L. The authors suggested that the phenomenon was nonspecific and likely associated with vasculopathy and decreased platelet number or function. Rehman et al. [3]. reported the case of a patient with a history of hypertension and type 2 diabetes who had a blood pressure of 231/136 mmHg after electroconvulsive therapy induction. Platelet count was 243 × 10^9^/L. Diabetes is known to increase capillary vulnerability in patients [4, 5]. Abdulla et al. [6] similarly reported Rumpel-Leede symptoms in a patient who underwent coronary stenting. The authors reported that the venous return was obstructed, but the arterial flow was normal, resulting in localized venous hypertension, which caused capillary rupture into the dermis, and a petechial rash. Rumple-Leede symptoms were reported by Rattka et al. [7] in a patient following coronary angiography; petechiae resolved in 2 days without further treatment. There were no signs of the Rumpel-Leede phenomenon during the subsequent outpatient follow-up.

We believe that, in contrast to the pathophysiological mechanisms of other acute dermatoses, all known cases of this Rumpel-Leede symptom have been reported with compression of the limb during treatment. We believe that the underlying mechanism for this phenomenon involves the compression of the patient's limb, leading to obstruction of venous return and thereby allowing the patient's capillaries to rupture into the dermis. Compression is usually required to prevent post-operative bleeding at surgical puncture sites. In some cases, anti-platelet agents are routinely administered to prevent thrombosis. In general, hypertension, hyperlipidaemia, and platelet dysfunction are the predisposing factors for Rumpel-Leede phenomenon. All of the cases reported so far have resolved spontaneously without treatment, implying that the phenomenon may be self-limiting. In patients undergoing coronary angiography, pre-procedure administration of anti-platelet agents is unavoidable. The ways to avoid the Rumpel-Leede symptoms may involve the perioperative patient modulation of platelet function and drug use, and the timing of compression hemostasis may require further clinical trials to avoid compression-induced venous reflux compression.

## Conclusion

we reported the case of a patient with hypertension and hyperlipidemia who had a routinely applied radial compression pressor after coronary angiography. The patient developed a petechial rash on the skin below the radial compression tourniquet, consistent with the findings of the Rumpel-Leede phenomenon. This phenomenon is relatively uncommon in clinical practice; however, clinicians should be watchful of these symptoms.

### Learning objectives

1. To better understand the possible mechanism of ecchymosis caused by the pressurizer and the underlying susceptibility factors that cause the rash.
2. To better understand a relatively rare phenomenon that clinicians must consider and try to avoid in current treatment programs.

## Data Availability

All data generated or analysed during this study are included in this published article.

## Abbreviations

PLT: Platelet
APTT: Activated partial thromboplastin clotting time
